# *Annona glabra* Flavonoids Act As Antimicrobials by Binding to *Pseudomonas aeruginosa* Cell Walls

**DOI:** 10.3389/fmicb.2016.02053

**Published:** 2016-12-21

**Authors:** Stanley de S. L. Galvão, Andrea de S. Monteiro, Ezequias P. Siqueira, Maria Rosa Q. Bomfim, Marcus Vinícius Dias-Souza, Gabriella F. Ferreira, Angelo Márcio L. Denadai, Áquila R. C. Santos, Vera Lúcia dos Santos, Elaine M. de Souza-Fagundes, Elizabeth S. Fernandes, Valério Monteiro-Neto

**Affiliations:** ^1^Centro de Ciências da Saúde, Universidade CEUMASão Luís, Brazil; ^2^Centro de Pesquisa René Rachou, Fundação Oswaldo Cruz-FIOCRUZBelo Horizonte, Brazil; ^3^Departamento de Microbiologia, Universidade Federal de Minas GeraisBelo Horizonte, Brazil; ^4^Departamento de Fármacia, Universidade Federal de Juiz de Fora, Campus Governador Valadares, Governador ValadaresBrazil; ^5^Departamento de Fisiologia e Biofisica, Universidade Federal de Minas Gerais, Belo Horizonte, Brazil; ^6^Departamento de Patologia, Universidade Federal do Maranhão, São LuísBrazil

**Keywords:** *Annona glabra* Pseudomonas aeruginosa, antimicrobial activity, flavonoids

## Abstract

*Pseudomonas aeruginosa* is an important pathogen in opportunistic infections in humans. The increased incidence of antimicrobial-resistant *P. aeruginosa* isolates has highlighted the need for novel and more potent therapies against this microorganism. *Annona glabra* is known for presenting different compounds with diverse biological activities, such as anti-tumor and immunomodulatory activities. Although other species of the family display antimicrobial actions, this has not yet been reported for *A. glabra*. Here, we investigated the antimicrobial activity of the ethyl acetate fraction (EAF) obtained from the leaf hydroalcoholic extract of *A. glabra*. EAF was bactericidal against different strains of *P. aeruginosa*. EAF also presented with a time- and concentration-dependent effect on *P. aeruginosa* viability. Testing of different EAF sub-fractions showed that the sub-fraction 32-33 (SF32-33) was the most effective against *P. aeruginosa*. Analysis of the chemical constituents of SF32-33 demonstrated a high content of flavonoids. Incubation of this active sub-fraction with *P. aeruginosa* ATCC 27983 triggered an endothermic reaction, which was accompanied by an increased electric charge, suggesting a high binding of SF32-33 compounds to bacterial cell walls. Collectively, our results suggest that *A. glabra*-derived compounds, especially flavonoids, may be useful for treating infections caused by *P. aeruginosa*.

## Introduction

Infection by *Pseudomonas aeruginosa*, a Gram-negative bacterium known to cause a variety of opportunistic infections in humans, can result in pneumonia, sepsis, meningitis, and urinary tract infections, as well as skin and soft-tissue infections (Wroblewska, 2006). The increasing prevalence of *P. aeruginosa* isolates that are resistant to almost all antimicrobials has raised concern among public health authorities in recent decades and highlights the importance of developing novel antimicrobial drugs active against this pathogen (Porras-Gomez et al., 2012). The development of resistance in *P. aeruginosa* is considered multifactorial, with mutations in several genes that encode eﬄux pumps, porins, penicillin-binding proteins, and chromosomal β-lactamases (Poole, 2011; Morita et al., 2014). All of these genes are involved in the resistance to distinct antibiotic classes, including penicillins, carbapenems, aminoglycosides, and fluoroquinolones (Ozer et al., 2009).

Natural compounds have been extensively studied as potential sources of antimicrobial compounds. Indeed, different natural compounds were suggested as antimicrobials against *P. aeruginosa* (Hayashi et al., 2014; Yang et al., 2015; Undabarrena et al., 2016); with some of them acting as eﬄux pump inhibitors in *P. aeruginosa*, thus contributing to reversal of resistance in these strains (Morita et al., 2016).

*Annona glabra* Linn is a tropical fruit tree native to Florida (United States of America), the Caribbean, and Central and South Americas. It has a wide distribution in Brazil, mainly in mangrove areas (Núñez-Elisea et al., 1999). *Annona glabra* is a potential source of compounds for cancer therapy, where an alcoholic seed extract has shown anticancer activity (Cochrane et al., 2008). In addition, a preliminary screening also demonstrated substantial antimicrobial activities for the hexane extract of *A. glabra* stem bark (Padmaja et al., 1995).

Antimicrobial activity was recently reported in other *Annona* species. The hexane and chloroform fractions obtained from *Annona vepretorum* leaves display antimicrobial activity against *Escherichia coli, Klebsiella pneumoniae*, and *Staphylococcus aureus* (Almeida et al., 2014). Additionally, Rinaldi and collaborators showed that the methanol, dichloromethane, and ethyl acetate fractions (EAFs) obtained from *Annona hypoglauca* stems inhibited the growth of *Staphylococcus aureus* and *Enterococcus faecalis* (Rinaldi et al., 2016).

Different compounds may confer antimicrobial properties for plants, with flavonoids being one of the major compounds to which these effects have been attributed. To date, compounds, such as annoglabayin and methyl-16 alpha-hydro-19-al-ent-kauran-17-oate (kaurane diterpenoids; Chen et al., 2004; Zhang et al., 2004) and squamosamides (Bao et al., 2015), have been identified and isolated from *A. glabra* fruits. However, little is known regarding the flavonoid content in this plant or their ability to function as antimicrobials.

Herein, we evaluated the antibacterial effects of the hydroalcoholic extract from *A. glabra* leaves against standard and clinical isolates of *P. aeruginosa* and characterized the antibacterial effects of the EAF. Additionally, the interactions between a flavonoid-rich sub-fraction (SF) of EAF (SF32-33) and *P. aeruginosa* was investigated by characterization of changes in the zeta potential (ZP) and enthalpy.

## Materials and Methods

### Plant

The stems, leaves, and fruits of *Annona glabra* Linn (Arecaceae) were collected at “Central do Maranhão,” Maranhão, Brazil (2°16′20.2′′ S, 44° 57′14.2′′ W). A voucher specimen (N° 01077) was deposited in the herbarium Ático Seabra of the Federal University of Maranhão, São Luís, Brazil.

### Preparation of Extracts from Leaves

Collected leaves were washed in running water for up to 5 min before being dried at room temperature. The dried leaves were triturated using a blender (Siemsen, model LI-1.5, São Paulo, Brazil) for 20 min to obtain a fine powder, and 300 g were extracted twice with 1000 ml ethyl alcohol (99.5%; Sigma–Aldrich, St. Louis, MO, USA) at room temperature, with a 5 days period between extractions. The mixture was filtered through cellulose filter paper (Whatman No. 4, GE Healthcare UK, Amersham, UK) and evaporated to dryness under reduced pressure using a rotary evaporator (Eyela N-1200BV-W, Tokyo, Japan) at 40°C. The residual solvent was removed in a vacuum centrifuge at 40°C to yield crude ethanol extracts of leaves.

### Preparation of the EAF from the Leaf Hydroalcoholic Extract of *A. glabra*

The EAF was obtained from the leaf hydroalcoholic extract of *A. glabra* as previously described (de Siqueira et al., 2014). For this, six grams of the ethanol extract were suspended in MeOH:H_2_O (4:1) using an ultrasonic bath (Elmasonic^®^ E 30H Elma, Sigen, Germany) and extracted successively by solvent-solvent partitioning. For concentration and mass determination, 10 ml of EAF was dried in a Speedvac system (Speed Vac Concentrator Sc110, Savant, Thermo Fisher Scientific Inc., Waltham, MA, USA) at 50°C for 60 h. For *in vitro* assays, EAF and SF stock solutions were prepared in dimethyl sulfoxide (DMSO; Sigma–Aldrich, St. Louis, MO, USA) at 50 mg/ml.

### Procedure for Gel Permeation Chromatography (GPC)

Ethyl acetate fraction SFs were obtained by GPC. For this, 3.15 g/ml of EAF was injected into GPC glass columns (Büchi column n° 17980), packaged with Sephadex LH-20 TM gel (GE Healthcare, USA), and eluted in 2 ml of 99% methanol (P.A., Merck, Darmstadt, Germany). For the mobile phase, the eluate was pumped at a flow rate of 480 ml/h, for 4 h and two hundred fractions were then collected (20 ml/tube).

### Thin Layer Chromatography (TLC) Analysis

Ethyl acetate fraction SFs were characterized by TLC analysis on pre-coated commercial silica gel plates G-60/F254 (0.25 mm, Merck, Darmstadt, Germany). Briefly, TLC plates were eluted using solvent mixtures in different proportions: (i) ethyl acetate:methanol:water (EtOAc:MeOH:H_2_O/80:11:5 v/v) for polar compounds or (ii) dichloromethane:methanol (DCM:MeOH/95:5 v/v) for non-polar compounds. High content flavonoid blottings were visualized under UV light at 360 nm after spraying the plates with a mixture (1:1) of an ethanol solution containing vanillin 1% w/v and sulfuric acid at 10% (v/v) or 2-aminoethyl diphenylborinate/polyethylene glycol 4000 (NP/PEG) solution in ethanol at 2% (w/v; de Siqueira et al., 2014). Sub-fractions with similar blotting profiles were pooled and analyzed in microbiological assays [Minimum inhibitory concentrations (MIC) and Minimum bactericidal concentrations (MBC)].

### Liquid Chromatography-High Resolution Mass Spectrometry (LC-HRMS) Analysis

The LC-HRMS (MSMS) analysis of the flavonoid-rich SF SF32-33 was performed on a Nexera UHPLC system (Shimadzu Corporation, Kyoto, Japan) hyphenated to a maXis ETD high resolution ESI-QTOF (ElectroSpray Ionization – Quadrupole Time of Flight) mass spectrometer (Bruker Daltonics, Bremen, Germany) and controlled by the Compass 1.5 software package (Bruker Daltonics, Bremen, Germany), according to a previously described method (de Siqueira et al., 2014). For analysis, SF32-33 (10 μg/ml) was dissolved in ACN:H_2_O (1:1 v/v) and injected into a Shimadzu Shim-Pack XR-ODS-III column (C18, 2.2 μm, 80 Å, 2.0 mm × 200 mm) at a flow rate of 200 μL/min. SF32-33 was then sequentially eluted with mixtures of water containing 0.1% formic acid (Solution A), followed by acetonitrile solution containing 0.1% formic acid (Solution B). For the application of Solution B, 10% was applied over a 10-min period, followed by a linear gradient (10–100%) for 40 min, and finally 100% for 5 min. Ion-source parameters were set to 500 V for end plate offset, 4500 V for capillary voltage, 2.0 bar nebulizer pressure, and 8.0 l/min dry gas flow at 200°C. Data-dependent precursor fragmentation was performed at collision energies of 30 eV. Ion cooler settings were optimized for an average sensitivity of 40–1000 *m*/*z* range using a solution of 10 mM sodium formate in 2-propanol/0.2% formic acid (1:1, v/v) as the calibration solution. Mass calibration was achieved by initial ion-source infusion of 20 μl calibration solution and post-acquisition recalibration of the raw data. Compound identification was performed by chromatographic peak dissection, with subsequent formula determination according to exact mass and isotope pattern (MS1) and database comparison of compound fragment spectra (MS2), as well as the comparison of compound fragment spectra and co-elution with standard compounds (Sigma–Aldrich, St. Louis, MO, USA). Sources of reference ESI fragmentation pattern spectra consisted of an in-house database of commercial or isolated and identified compounds, as well as the public spectra database MassBank (Horai et al., 2010).

### Antimicrobial Activity Assays

Antibacterial activity assays were evaluated using the following microorganisms: *P. aeruginosa* ATCC 27983 (kindly donated by the Instituto Nacional de Controle de Qualidade em Saúde – da Função Instituto Osvaldo Cruz, INCQS–FIOCRUZ, Rio de Janeiro, Brazil) and 10 multi-resistant *P. aeruginosa* clinical strains from the culture collection of the laboratory (**Table 1**, first column). Minimum inhibitory concentrations were determined using the broth microdilution method, as described in CLSI M07-A10 (CLSI, 2015), with minor modifications. For this, different concentrations of EAF or SF32-33 (0.05–1024 μg/ml) were incubated with 100 μl of Mueller–Hinton broth (MHB, Difco, Detroit, MI, USA) in 96-well plates. Vehicle (1% DMSO)-treated wells were used as negative controls. Then, a bacterial inoculum (5 × 10^5^ CFU/mL) was added into the wells. Sterility controls were included for each assay, and tests were performed on three occasions in triplicate. Ciprofloxacin-treated wells (Sigma–Aldrich, St. Louis, MO, USA) were used as positive controls (0.05–1024 μg/ml). The microdilution plates were incubated under aerobic conditions at 35°C for 24 h. The MIC was defined as the lowest concentration of EAF or SF32-33 that completely inhibited the visible growth of microorganisms.

**Table 1:** Antimicrobial activity of the leaf hydroalcoholic extract and the ethyl acetate fraction (EAF) of *Annona glabra* on *Pseudomonas aeruginosa* strains.

Bacterial strains	Concentration (μg/ml)			
	**Hydroalcoholic extract**		**EAF**	
	**MIC**	**MBC**	**MIC**	**MBC**

ATCC 27853	1024	2048	8	16
P1C	1024	2048	8	16
P2C	1024	2048	8	16
P5C	1024	2048	8	16
P18C	1024	2048	8	16
P27C	1024	2048	8	16
P32C	1024	2048	4	4
P110c	1024	2048	8	16
P113C	1024	2048	8	16
P146C	1024	1024	4	8
P165C	1024	2048	8	16

*Minimum inhibitory concentrations (MIC) and minimum bactericidal concentrations (MBC) were calculated and expressed in μg/ml. Assays were performed in duplicate on three different experiments*.

Minimum bactericidal concentrations were evaluated soon after determining the MIC. For this, 100 μl of culture medium was removed from each well with no visible growth and transferred to Mueller–Hinton agar plates. Plates were then incubated at 35°C for 24 h. The MBC was considered as the lowest concentration that either totally prevented growth or resulted in a ≥99.9% decrease in the initial inoculum (i.e., a 3 log_10_ reduction in CFU/ml) upon subculture. Bacteriostatic action was defined as a ratio of MBC to MIC that is >4. Otherwise, a lower MBC to MIC ratio was defined as bactericidal action (Pankey and Sabath, 2004). All tests were performed in triplicate.

### Time-Kill Assays

The time-dependent effects of SF32-33 on *P. aeruginosa* viability were evaluated by measuring the reduction in the numbers of CFU per milliliter for 3–24 h. Briefly, SF32-33 (2.0–64 μg/ml) was added to each well and incubated with 10^4^ CFU/ml of *P. aeruginosa* ATCC 27983 for 3–24 h at 35°C. A 100-μl aliquot of bacterial inoculum was removed from the microtiter plates containing SF32-33 at different intervals until 24 h, and were serially diluted in sterile saline, before plating onto MacConkey agar (BBL^TM^ MacConkey Agar, BD, Sparks, MD, USA) plates for colony count determinations. The plates were incubated at 35°C for 24 h prior to colony counting. The results were expressed as the percentage of *P. aeruginosa* in relation to the negative control wells (vehicle-treated) for each time analyzed. The data represent the mean values of three independent experiments in duplicate assays. Vehicle-treated wells were used as controls.

### Effect of Flavonoids on Zeta Potential of Bacterial Cell Surfaces

To evaluate whether the SF32-33 interacts with the *P. aeruginosa* cell walls, *P. aeruginosa* ATCC 27983 was grown in Tryptic Soy Broth (Bacto^TM^ Tryptic Soy Broth, TSB, BD, Sparks, MD, USA) for 18 h at 35°C, as previously described (Soon et al., 2011). Cells were then washed three times with sterile phosphate buffered saline (PBS, pH = 7.2) and centrifuged at 5,000 ×*g* for 10 min at 4°C. The supernatant was discarded, and the cells were resuspended in 10 ml of KCl solution (10 mM) and vortexed for 1 min.

The ZP was measured to determine the ability of SF32-33 to modify the electrical charge of the *P. aeruginosa* cell walls. ZP measurements were conducted at 25°C using a 64-channel Zetasizer Nano ZS system (Malvern) at 633 nm (red Leisure) and Malvern Laser Doppler Velocimetry patterns coupled with M3-PALS (Phase Analysis Light Scattering). Briefly, 3 ml of a *P. aeruginosa* cell suspension containing 1 × 10^8^ CFU/ml was incubated with 10 μl of a SF32-33 stock solution of 23 μg/ml into a 25 ml glass container. Then, 1 ml of the mixture was transferred into a plastic cuvette (Cell-Folded Capillary DTS1060; Malvern). Titration (0–1.5 μg/ml) was performed 20 times by manual injection of 10 μl of the mixture. For each titration, the solution was transferred to the same cuvette, and the liquid charge was recorded in millivolts (mV), which was considered as the ZP. Each titration was repeated 10 times, and the results were expressed as the average of ten repetitions for each titration.

### Isothermal Titration Calorimetry Assays

Isothermal titration calorimetry (ITC) assays were performed to determine the thermodynamic parameters of interaction between the SF32-33 and *P. aeruginosa* ATCC 27853 cells in suspension. The experiments were performed in duplicate using a VP-ITC microcalorimeter at 25°C (MicroCal Company, Northampton, MA, USA). The titration consisted of 50 5-μL successive automated injections (at intervals of 300 s each) of an SF32-33 stock solution (23 μg/ml) into a cuvette containing 1.5 ml of *P. aeruginosa* suspension (1 × 10^8^ CFU/ml). The SF32-33 concentrations ranged from 0 to 3.28 μg/ml. Corrections of concentrations, as well as the integration of the heat flow peaks involved in specific enthalpies of interaction (Δ_inj_H° in kcal/μg of SF32-33), were made using MicroCal Origin 7.0 software for ITC (Microcal, Northampton, MA, USA). Injections of SF32-33 into a cuvette charged with 1.5 ml of solvent only were used as the control in order to determine possible release of energy during titration. All solutions were prepared in 0.9% saline in Milli-Q purified water (pH = 7.0). The results were expressed as the difference of enthalpy between the solvent and SF32-33 itself (Δ_inj_H°).

### Statistical Analysis

The data obtained in the time-kill and cytotoxicity assays were analyzed with GraphPad Prism (version 5.0; GraphPad Software, Inc. San Diego, CA, USA) using the Mann–Whitney non-parametric tests with a confidence interval of 95%. Differences between treated and control groups were considered significant for any *p*-value < 0.05.

## Results

### Characterization of the Antimicrobial Effects of the Leaf Hydroalcoholic Extract and EAF of *A. glabra*

Qualitative analysis of the hydroalcoholic extract of *A. glabra* showed a high content of flavonoids. A similar profile was found for the EAF when it was analyzed by TLC, especially for the SF32-33 fraction. Evaluation of the antimicrobial effects of *A. glabra* showed a MIC of 1024 μg/ml for the hydroalcoholic extract when tested against the *P. aeruginosa* clinical isolates and ATCC 27853 (**Table 1**). An MBC of 2048 μg/ml was observed for all the tested bacteria, except for one of the clinical isolates (P146c) in which the MBC was 1024 μg/ml (**Table 1**). EAF was highly effective against *P. aeruginosa*, presenting an MIC of 4–8 μg/ml and an MBC of 8–16 μg/ml (**Table 1**). MIC evaluation for different SFs showed that SF32-33 (4 μg/ml) was the most effective against *P. aeruginosa*, followed by SF18-19, which presented an MIC of 64 μg/ml (**Table 2**). All the other SFs tested exhibited MICs ranging from 256 to 512 μg/ml (**Table 2**). LC-HRMS analysis confirmed that SF32-33 is a flavonoid-rich SF containing (-) epicatechin, fisetin, quercetin, rutin, hyperosid, isoquercitrin, quercitrin, kaempferol 7-neohesperidoside, nicotiflorine, and naringenin (**Table 3**). Ciprofloxacin, a commercially available antimicrobial, exhibited an MIC and MBC of 32 and 64 μg/ml, respectively, for the multi-resistant clinical strain of *P. aeruginosa* P2C. Therefore, both SF32-33 and ciprofloxacin were able to significantly reduce *P. aeruginosa* growth, with reductions of 5.92 and 5.87 log CFU/ml, respectively.

**Table 2:** Antimicrobial activity of different ethyl acetate fraction (EAF) sub-fractions (SF) of *A. glabra* on *P. aeruginosa* strains.

Sub-fractions	Concentration (μg/ml)	
	ATCC 27853	P2C
SF 1-15	512	512
SF 16-17	512	512
SF 18-19	64	64
SF 20-22	512	512
SF 23-24	512	512
SF 25-26	512	512
SF 27-28	512	512
SF 29-31	512	512
SF 32-33	4	4
SF 34-36	512	512
SF 37-40	256	256
SF R	512	512

*Minimum inhibitory concentrations were calculated and expressed in μg/ml. Assays were performed in duplicate on three different experiments*.

**Table 3:** Liquid chromatography-high resolution mass spectrometry (LC-HRMS) analysis of SF32-33.

Compound	Rt/min	*m/z* [M+H]^+^	Formula	Fragment ions (abundance)	Identification method	Fit score (purity)
(-) Epicatechin	9.4	291.0867	C_15_ H_14_O_6_	291.0867 (100)291.1497 (2.5)	Standard/in-house DB	990
Rutin	14.7	305.0661, 611.1612	C_15_ H_12_O_7_ C_27_H_31_O_16_	153.0183 (100) 185.0605 (37.8) 201.0571 (19.9)	Standard/in-house DB	999
Hyperoside	14.9	465.1037	C_21_ H_20_ O_12_	303.0502 (100)303.1152 (2.3)303.4847 (1.8)	Standard/in-house DB	999
Quercetin	15.7	303.0503, 435.0929	C_15_H_10_O_7_ C_20_H_18_O_11_	303.0501 (100)303.1152 (2.7)303.4851 (1.9)	Standard/in-house DB	990
Fisetin	16.3	287.0554	C_15_H_10_O_6_	287.0547 (100) 288.0580 (13.1)289.0602 (2.1)	Standard/in-house DB	928
Quercitrin	17.0	303.0502,449.108	C_15_H_10_O_7_C_21_ H_20_O_11_	303.0500 (100) 304.0533 (15.8)305.0558 (2.6) 449.1082 (12.8)	Standard/in-house DB	931
Kaempferol-7-neohesperidoside nicotiflorine	19.0	595.1460	C_27_H_30_O_15_	595.1460 (100) 596.1505 (63.3) 597.1533 (22.4) 625.1564 (25.3)	Standard/in-house DB	820
Naringenin	19.9	773.1933, 273.0762	C_15_H_12_O_5_	773.1933 (100)773.2955 (2.8) 774.1968 (37.6)153.0184 (100)154.0218 (6.3)	Standard/in-house DB	990

### Time-Kill Assays

**FIGURE 1:**
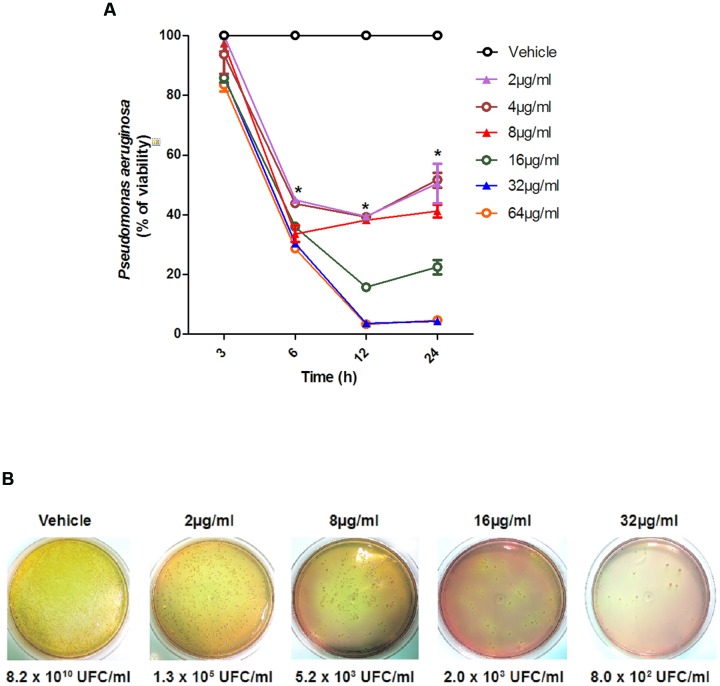
**Time-dependent effect of SF32-33 on *Pseudomonas aeruginosa* ATCC 27853 survival. (A)** Percentage of *P. aeruginosa* viability in relation to the negative control wells (vehicle-treated) at different time points (3–24 h). **(B)** Representative panels of *P. aeruginosa* growth in CFU/ml following incubation with SF32-33 for 12 h. Assays were performed three times in duplicate. The limit of detection in the assay was of 10^3^ CFU/ml. ^∗^*p* < 0.05; differs from the vehicle-control.

To evaluate whether the effect of SF32-33 on *P. aeruginosa* was time- and/or dose-dependent, we performed a time-kill assay. **Figure 1** shows that SF32-33 presented a dose- and time-dependent effect on *P. aeruginosa* ATCC 27853 where growth inhibition was maximal at 24 h post-incubation (96% inhibition; *p* < 0.005).

### Effect of SF32-33 toward the Zeta Potential of *P. aeruginosa*

**FIGURE 2:**
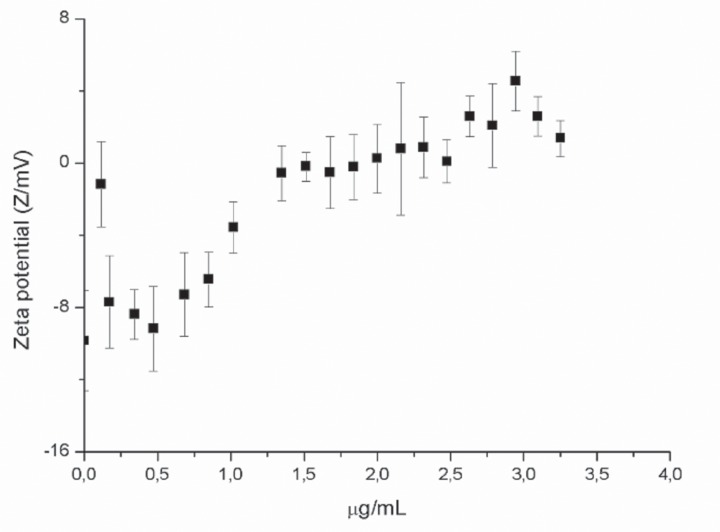
**Effect of flavonoids on the zeta potential (ZP) of *P. aeruginosa* ATCC 27983**. ZP was measured in 10 mM KCl expressed in mV as a function of SF32-33 concentration. Each point represents an average of 10 repetitions for each titration.

Zeta potential measurements were performed in order to investigate the ability of SF32-33 to interact with the cell wall surface of *P. aeruginosa* ATCC 27853. The results showed that by incubating *P. aeruginosa* with cumulative concentrations of SF32-33 (up to 1.5 μg/ml), there was a shift in the ZP from a negative to positive charge (-8.21 to +2.95 mV; **Figure 2**).

### Isothermal Titration Calorimetry Assays

**FIGURE 3:**
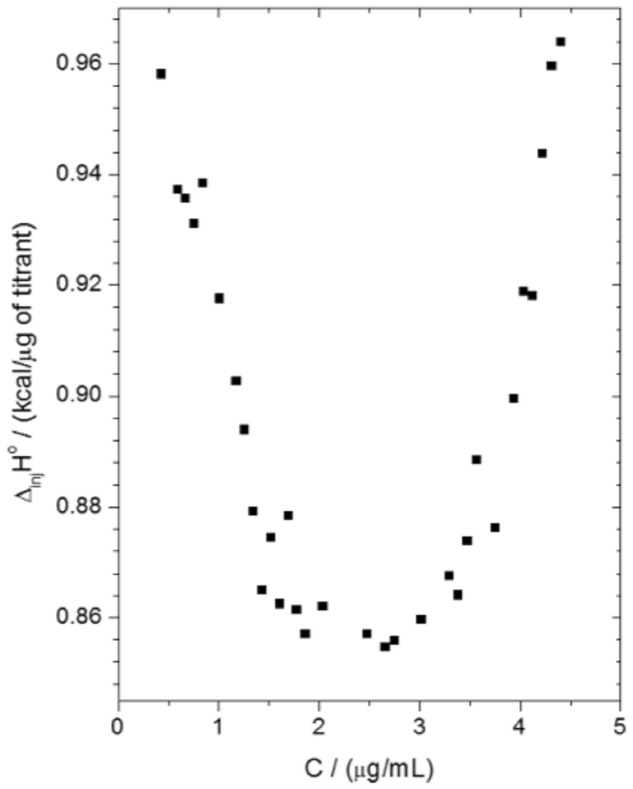
**Isothermal titration calorimetry (ITC) result of the interaction thermodynamics between SF32-33 and *P. aeruginosa* ATCC 27853**. The titration consisted of 50 5-μL successive automated injections (at intervals of 300 s each) of SF32-33 stock solution (23 μg/ml) into a cuvette containing 1.5 ml of *P. aeruginosa* suspension (1 × 10^8^ CFU/ml). The results are expressed as the difference of enthalpy between the solvent and SF32-33 itself (Δ_inj_H° in kcal/μg).

Isothermal titration calorimetry assays were performed in order to analyze the thermal profile of the interaction between SF32-33 and *P. aeruginosa* ATCC 27853. The data depicted in **Figure 3** demonstrate that the incubation of cumulative concentrations of SF32-33 (0–3.28 μg/ml) with *P. aeruginosa* resulted in an endothermic reaction, as indicated by the graph of molar enthalpy of titrant – Δ_inj_H° (in kcal/μg of titrant), against distinct concentrations of SF32-33 in μg/ml. Furthermore, the observed parabolic profile obtained suggests that the interaction between SF32-33 and *P. aeruginosa* may occur through different mechanisms.

## Discussion

Although antimicrobial activities were previously reported for other *Annona* species, little is known on *A. glabra* ability to inhibit bacterial growth and/or viabilty. To the best of our knowledge we present the first evidence on that the EAF obtained from the leaf hydroalcoholic extract of *A. glabra* presents a potent antimicrobial activity against *P. aeruginosa*. We found that this activity is especially related to SF32-33, a flavonoid-rich SF of EAF. The antimicrobial actions of SF32-33 are due to its ability to interact with the bacterial cell wall surface, resulting in an endothermic reaction. These results suggest the existence of a strong binding event between SF32-33 and *P. aeruginosa*.

Analysis of the MIC and MBC of the hydroalcoholic extract, EAF, and SF32-33 demonstrated that the hydroalcoholic extract was not as effective as the EAF and SF32-33 in inhibiting bacterial growth and survival. By comparison, SF32-33 was the most effective against *P. aeruginosa*, presenting the lowest MIC and MBC. The effect of SF32-33 on *P. aeruginosa* viability was further investigated in a time-kill assay. SF32-33 had a dose- and time-dependent effect on *P. aeruginosa* ATCC 27853. Indeed, these effects were observed as soon as 6 h post-incubation of SF32-33 and lasted for 24 h. Although antibacterial activity has been attributed to fractions obtained from other plants of the *Annona* genus (*A. vepretorum and A. hypoglauca*), their MIC and MBC were much higher (≤100 mg/ml; Almeida et al., 2014; Rinaldi et al., 2016) in comparison to those observed for the EAF of *A. glabra* in our study (≤16 μg/ml).

The TLC and LC-HRMS results indicated that SF32-33 is rich in flavonoids. Whilst the other fractions of the extract (methanol, hexane, and dichloromethane) displayed low content of flavonoids (data not shown), partitioning of the extract with ethyl acetate allowed a greater enrichment in flavonoids. The antibacterial effects of flavonoids have been widely reported in the literature. Indeed, these effects have been attributed to their chemical structure, considering the number and positions of methoxyl and hydroxyl groups in the core C3 (Wu et al., 2013), which confer an intrinsic electrical charge and high hydrophobicity to these compounds. These characteristics favor increased membrane fluidity in the bacterial cell (Tsuchiya and Iinuma, 2000; Wu et al., 2013). In a recent study using a structure-activity relationship model, flavonoids were shown to affect *Escherichia coli* viability by damaging its outer membrane (Fang et al., 2016). Considering the high hydrophobicity of both flavonoids and the *P. aeruginosa* cell wall, it is possible that SF32-33 has an effect on *P. aeruginosa* viability and growth due to a strong hydrophobic interaction.

By performing an LC-HRMS analysis, it was possible to identify different flavonoids in the SF32-33, including (-)-epicatechin, fisetin, quercetin, rutin (quercetin glycoside), hyperosid, isoquercitrin, quercitrin, kaempferol 7-neohesperidoside, nicotiflorine, and naringenin. To our knowledge, we present the first evidence of the compounds found in the EAF of *A. glabra*. Many of them have already been shown to present antimicrobial actions against different bacteria, but little is known of their ability to inhibit *P. aeruginosa* growth/viability.

The structure of (-) epicatechin is composed of two aromatic rings linked by an oxygen heterocycle with a 4-hydroxyl group (Fraga and Oteiza, 2011). Epicatechins are flavan-3-ols, a subfamily of flavonoid polyphenolic compounds that are abundant in grape seeds and peels, green tea, nuts, and berries. Epicatechins, such as (-)-epicatechin gallate, inhibit the expression of virulence genes associated with secretion of proteins in *Staphylococcus aureus* (Shah et al., 2008). To the best of our knowledge, few reports have characterized epicatechins isolated from the leaves of plants of the *Annonaceae* family, and even fewer reports have been published on their antibacterial actions (Lage et al., 2014). Fisetin (3,3′,4′,7-tetrahydroxyflavone), another biologically active flavonoid, was also identified in SF32-33. Although this compound does not present antimicrobial properties, it has been suggested as a possible anti-virulence approach. Indeed, fisetin inhibits the action of listeriolysin O, a hemolytic toxin produced by *Listeria monocytogenes* whose main function is to facilitate bacterial replication and evasion from the host immune system (Wang et al., 2015). Another compound identified in SF32-33 is quercetin (3,3′,4′,5,7-pentahydroxyflavone). Reports have shown a potential use for this compound when combined with either epigallocatechin-3-gallate c, against drug-resistant *Mycobacterium tuberculosis* (Dey et al., 2015), or oxacillin, causing damage in the cell walls of multi-resistant *Staphylococcus aureus* (Amin et al., 2015). It is possible that all the compounds identified herein act synergistically, contributing to the antimicrobial effects of SF32-33.

We next assessed the ability of SF32-33 to interact with the cell wall of *P. aeruginosa*. We found that incubation of SF32-33 with *P. aeruginosa* caused a shift in the ZP from a negative to positive charge. We suggest that this interaction is mediated by the flavonoids present in the SF32-33. In fact, flavonoids have been reported to interact with *E. coli* cell wall proteins, changing their structure, and this has been associated with an increase in ZP (Wu et al., 2013). We additionally performed ITC assays and found that the incubation of SF32-33 with *P. aeruginosa* promotes an endothermic reaction. We suggest that this entropic reaction is a result of the hydrophobic interactions between flavonoids in the SF32-33 and the bacterial cell wall surface, leading to a high extension of desolvation of ions and water molecules. These data taken together suggest that the interactions between SF32-33 and the *P. aeruginosa* cell wall account for the antibacterial actions of this *A. glabra* SF.

Collectively, our results show that flavonoids obtained from the EAF of *A. glabra* are effective against *P. aeruginosa* due to their ability to interact with the bacterial cell wall, and thus, may represent an alternative treatment for *P. aeruginosa* infection.

## AuthOr Contributions

SG collected the plants, prepared the extracts, performed the experiments, and wrote the manuscript; AM and MB collaborated on antimicrobial assays and data interpretation; GF, ES, ÁS, AD, MD-S, VS, and ES-F performed the chemical characterization, ZP contributed to ITC determination assays and data interpretation; EF contributed to statistical analysis, data interpretation, and critically revised the manuscript; VM-N contributed to the conception of the study, study design and coordination and critically revised the manuscript. All authors have read and approved the final version for publication.

## Conflict of Interest Statement

The authors declare that the research was conducted in the absence of any commercial or financial relationships that could be construed as a potential conflict of interest.
